# Permanent Pacemaker Timing Dilemma in Recovered Lymphocytic Myocarditis

**DOI:** 10.1016/j.jaccas.2025.106239

**Published:** 2025-12-02

**Authors:** Keita Tashiro, Takeshi Kashimura, Ryohei Sakai, Takayuki Inomata

**Affiliations:** Department of Cardiovascular Medicine, Niigata University Graduate School of Medical and Dental Sciences, Niigata, Japan

**Keywords:** acute heart failure, blood tests, bradycardia, cardiac magnetic resonance, cardiac pacemaker, circulation, echocardiography, electrophysiology, hemodynamics, imaging

## Abstract

**Background:**

The timing of permanent pacemaker implantation in myocarditis-related complete heart block remains challenging because no established criteria exist for reversibility of the conduction system.

**Case Summary:**

A previously healthy 50-year-old Asian woman presented with lymphocytic myocarditis complicated by cardiogenic shock and complete atrioventricular block (CAVB). Despite successful hemodynamic stabilization with Impella CP support and complete recovery from left ventricular dysfunction, CAVB persisted. A permanent pacemaker was implanted on day 28. Afterward, cardiac enzymes and left ventricular function remained normal, and follow-up cardiac magnetic resonance showed no myocardial edema or late gadolinium enhancement. However, CAVB remained.

**Discussion:**

This case demonstrates dissociation between cardiac recovery of systolic function and conduction system recovery in myocarditis. Despite complete normalization of all conventional indicators, conduction impairment persisted, highlighting the complexity of permanent pacemaker timing decisions.

**Take-Home Messages:**

Cardiac functional recovery does not always correlate with conduction system recovery in myocarditis. Comprehensive evaluation is essential for permanent pacemaker timing decisions.

## Visual Summary

**Figure:**
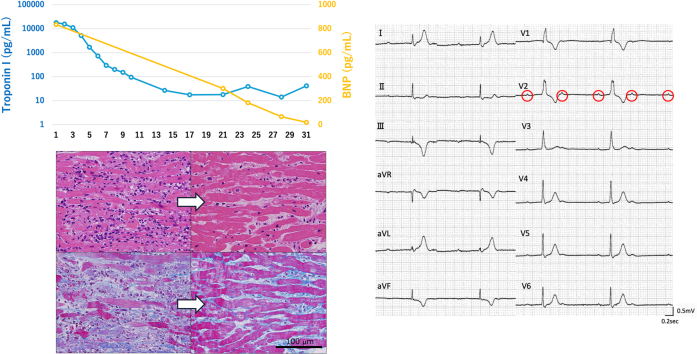

**Visual Summary:**
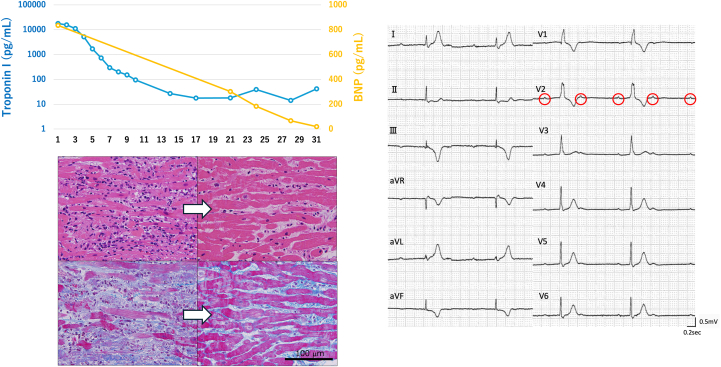
Lymphocytic Myocarditis with Permanent Complete Heart Block, Despite Functional Complete Recovery Arrows in pathology images: indicate the progression from acute phase (left) to recovery phase (right) with the same staining method. Red circles in ECG: indicate P-waves to demonstrate atrioventricular dissociation in complete heart block. BNP = brain natriuretic peptide.

## History of Presentation

A previously healthy Asian woman in her 50s developed fever, nausea, abdominal pain, and diarrhea 5 days before admission (day −5). Three days later (day −2), she began experiencing chest pain and dyspnea, prompting her to seek medical attention at a local hospital on day 0. Initial evaluation suggested acute myocarditis, and she was subsequently transferred to our institution. On arrival, she was alert and oriented with the following vital signs: blood pressure of 113/80 mm Hg, heart rate of 120 beats/min, oxygen saturation of 99% on room air, and body temperature of 37.0 °C. Physical examination revealed cool extremities and no heart murmurs; however, bilateral radial artery pulsations were weak.

**Take-Home Messages**

• Cardiac functional recovery does not always correlate with conduction system recovery in myocarditis.
• Comprehensive evaluation is essential for permanent pacemaker implantation decisions in myocarditis-related atrioventricular block.

## Past Medical History

The patient had no significant past medical history and was not taking any medications. There was no family history of cardiac disease or sudden death. She consumed alcohol occasionally, never smoked, and denied illicit drug use.

## Differential Diagnosis

The differential diagnosis included acute myocarditis, acute coronary syndrome, and Takotsubo cardiomyopathy as potential causes of acute heart failure. Additional considerations included pulmonary embolism, pneumothorax, and sepsis secondary to infectious disease. Comprehensive evaluation including blood tests, electrocardiography, echocardiography, cardiac catheterization, and endomyocardial biopsy was planned for definitive diagnosis of acute myocarditis and exclusion of alternative etiologies.

## Investigations

**Figure 1:**
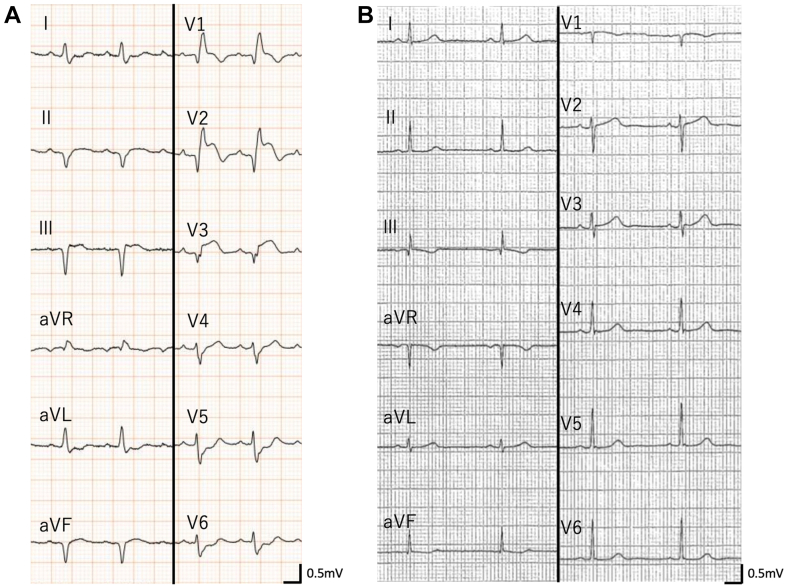
Electrocardiograms Showing Newly Acquired Conduction Abnormalities (A) Electrocardiogram on admission that revealed bifascicular block, ST-segment elevation in leads V_1_ to V_3_, and ST-segment depression in leads V_5_ and V_6_. (B) Previous electrocardiogram from health checkup 6 months prior revealed no abnormalities.

Laboratory data revealed significantly elevated cardiac enzymes (creatine kinase: 537 U/L, creatine kinase-MB: 104 U/L, troponin I: 17,848.4 pg/mL), and brain natriuretic peptide (BNP) was 834.6 pg/mL. Liver enzymes (aspartate aminotransferase: 118 U/L, alanine aminotransferase: 72 U/L) and lactate (2.1 mmol/L) were also elevated, but renal function (creatinine: 0.58 mg/dL, blood urea nitrogen: 17 mg/dL) remained normal. Electrocardiography revealed bifascicular block, ST-segment elevation in leads V_1_ to V_3_, and ST-segment depression in leads V_5_ and V_6_ (Figure 1A). Previous electrocardiographic findings were normal (Figure 1B), indicating newly acquired conduction impairment. Echocardiography revealed severe diffuse hypokinesis with an ejection fraction of 30%. The left ventricular wall was mildly thickened with no significant valvular abnormalities (Video 1).

**Figure 2:**
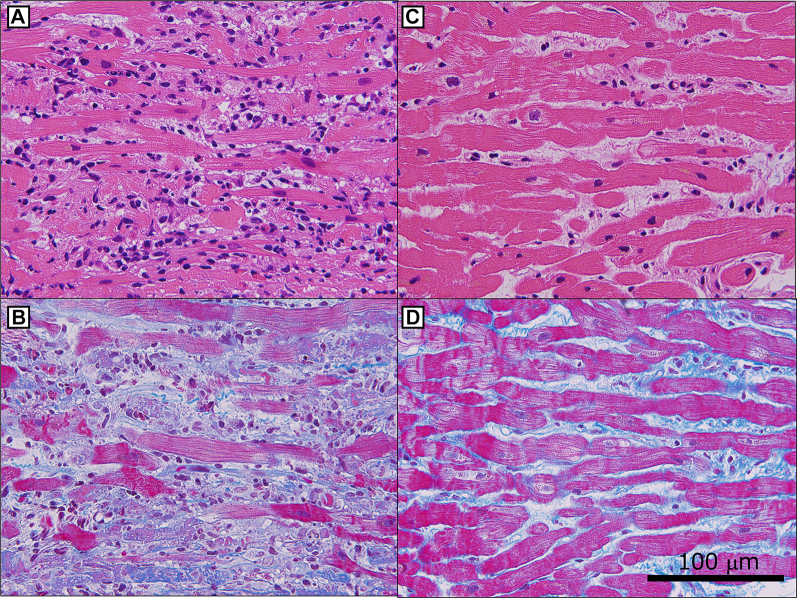
Endomyocardial Biopsy Findings in Acute and Recovery Phases (A and B) Endomyocardial biopsy on admission. (A) Hematoxylin-eosin stain revealed lymphocytic infiltration, myocardial fiber disruption, and loss. (B) Masson-trichrome stain revealed slight fibrosis. (C and D) The second endomyocardial biopsy on day 22. (C) Hematoxylin-eosin stain revealed reduced lymphocytic inflammation. Myocardial edema partially remained, but most cardiomyocytes were well preserved. (D) Masson-trichrome stain revealed interstitial fibrosis.

Right heart catheterization demonstrated elevated mean pulmonary artery wedge pressure (19 mm Hg) and pulmonary artery pressure (24/16 [20] mm Hg) with decreased cardiac output (Fick cardiac output: 2.21 L/min, cardiac index: 1.46 L/min/m^2^) and reduced pulmonary artery pulsatility index (0.89), suggesting cardiogenic shock due to biventricular failure. Complete atrioventricular block CAVB) developed during this catheterization procedure. Coronary angiography revealed no significant stenosis. Subsequently, endomyocardial biopsy was performed, revealing lymphocytic infiltration with myocardial fiber disruption and loss (Figures 2A and 2B).

## Management

We diagnosed lymphocytic myocarditis complicated by cardiogenic shock and conduction impairment based on pathologic and clinical findings. Dobutamine was introduced to support biventricular failure, and an Impella CP (Abiomed, Inc) was deployed to support left ventricular function and provide anti-inflammatory effects through ventricular unloading.^1^ Additionally, temporary pacing was initiated for conduction impairment. We prepared venoarterial extracorporeal membrane oxygenation in case of further deterioration of right ventricular failure.

**Figure 3:**
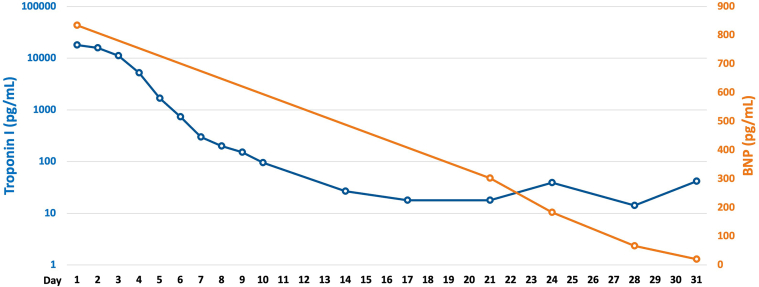
Serial Changes in Troponin I and BNP Levels Both biomarkers normalized rapidly. BNP = brain natriuretic peptide.

Fortunately, hemodynamics improved gradually. Impella removal was achieved on day 6, and dobutamine was discontinued on day 8. Cardiac enzymes normalized rapidly (Figure 3), and cardiac function improved progressively (Videos 2 and 3). Despite normalization of hemodynamics, cardiac enzymes, and function, CAVB persisted. The patient remained completely dependent on temporary pacing, precluding cardiac magnetic resonance (CMR) acquisition. We decided to perform a second endomyocardial biopsy on day 22 to evaluate myocardial edema and irreversible damage pathologically. It revealed reduced lymphocytic inflammation. Myocardial edema partially remained, and some areas revealed fibrotic replacement. However, most cardiomyocytes were well preserved (Figures 2C and 2D). This demonstrated pathologic improvement without atrioventricular conduction recovery.

Despite gradual clinical improvement and no recurrent exacerbation, CAVB persisted. Permanent pacemaker implantation was performed on day 28. The postoperative course was uneventful, and she was discharged on day 33.

## Outcome and Follow-Up

**Figure 4:**
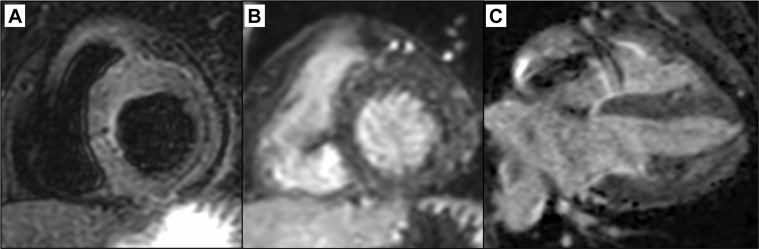
Cardiac Magnetic Resonance in the Chronic Phase (A) T2-weighted images revealed no hyperintense areas indicating myocardial edema. (B and C) There was no late gadolinium enhancement throughout the myocardium including conduction areas.

**Figure 5:**
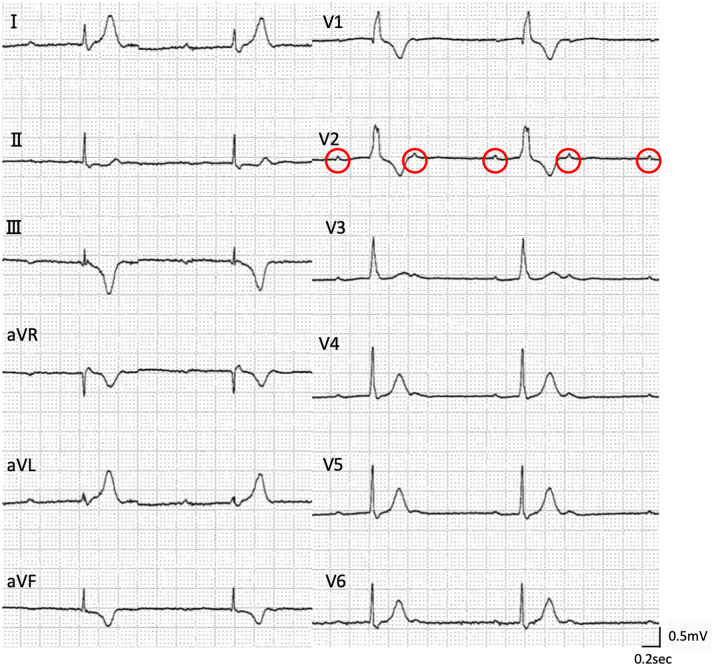
Electrocardiogram in the Chronic Phase Complete atrioventricular block persisted. The red circles indicates P-wave.

At the follow-up visit on day 78, she was asymptomatic. CMR revealed no myocardial edema on T2-weighted imaging and no late gadolinium enhancement (LGE) (Figure 4). Biomarkers remained normal (troponin I: <10 pg/mL, BNP: 15.5 pg/mL). Left ventricular function remained normal on echocardiography (Video 4), but she remained pacemaker-dependent. Persistent CAVB was confirmed by temporarily lowering the pacing rate (Figure 5).

## Discussion

Atrioventricular block is a rare complication of acute myocarditis, occurring in approximately 1.7% of cases.^1^ Patients with high-grade atrioventricular block demonstrate significantly worse prognosis.^2^ Atrioventricular block is an uncommon complication in typical lymphocytic myocarditis and occurs more frequently in specific conditions such as giant cell myocarditis, sarcoidosis, and Lyme disease.^3^^,^^4^ The presence of atrioventricular block suggests specific myocarditis subtypes and worse prognosis. Therefore, aggressive evaluation including endomyocardial biopsy is essential for clinical and pathologic diagnosis.

Cases with persistent conduction abnormalities despite complete recovery of myocardial function are very rare. The cardiac conduction system and working myocardium consist of fundamentally different cellular components, potentially resulting in distinct inflammatory responses, repair processes, and fibrotic mechanisms. However, atrioventricular block remains a relatively uncommon complication of myocarditis,^1^ suggesting that the conduction system is not necessarily more vulnerable to inflammation than the working myocardium. Rather, the important factor may be the anatomic characteristics of the conduction system. The cardiac conduction system has an extremely delicate structure compared with the working myocardium and must function as continuous conducting fibers. Therefore, tissue damage that would be minor and functionally insignificant to the working myocardium could potentially disrupt the conducting pathway and cause complete functional impairment of the conduction system. In our case, such focused microscopic damage around the conduction system may have occurred.

CMR is an established imaging modality for diagnosing and monitoring acute myocarditis.^5^ Assessment of myocardial edema on T2-weighted images and myocardial fibrosis on LGE are important. In immune checkpoint inhibitor–induced myocarditis, combining fluorodeoxyglucose positron emission tomography with CMR demonstrated promise for assessing irreversibility.^6^ However, this approach may not apply to infectious myocarditis due to different mechanisms and courses. Furthermore, temporary pacemakers are magnetic resonance imaging–incompatible; therefore, CMR cannot be performed when conduction is completely pacing-dependent. These constraints pose practical clinical challenges in imaging evaluation, particularly in myocarditis complicated by arrhythmia. Additionally, acute-phase LGE does not always reflect irreversibility. LGE detected in acute-phase myocarditis completely disappeared in 10% of cases during the chronic phase.^7^ Acute-phase LGE sometimes indicates contrast leakage due to inflammatory cell infiltration or interstitial edema, making it inappropriate for determining irreversibility in the acute phase. In our case, CAVB persisted despite the absence of LGE in the chronic phase, suggesting limitations in CMR detection sensitivity.^8^ However, chronic-phase CMR can detect fibrosis when present and contributes to differentiating chronic active myocarditis, making this examination important.

There are no established criteria suggesting the optimal timing for permanent pacemaker implantation in myocarditis-related atrioventricular block. Complete heart block may resolve within approximately 1 week if reversible,^9^ but no clear timeline exists. Minor inflammation may persist for 4 to 8 weeks,^10^ further complicating decision-making. We think that assessing inflammatory activity is crucial. When cardiac enzymes remain elevated, cardiac dysfunction persists, or myocardial edema is present, inflammation should be considered active. Quelling this inflammation may potentially improve conduction impairment; therefore, permanent pacemaker implantation should not be rushed. Early permanent pacemaker implantation may result in placing an unwanted foreign body if conduction recovers afterward. Conversely, in cases like ours where cardiac enzymes normalized rapidly and cardiac function improved, inflammation appears to have subsided. CMR is useful as a noninvasive method for multifaceted assessment of inflammatory activity. However, it is important to note that in the acute phase, CMR should be used to assess inflammatory activity (edema) rather than irreversibility (fibrosis), as previously mentioned. We overcame the hurdle of being unable to perform acute-phase CMR through repeated endomyocardial biopsies. However, it is crucial to note that endomyocardial biopsy can potentially cause iatrogenic conduction abnormalities and provides only focal tissue assessment rather than comprehensive myocardial evaluation. In complex cases, if minimal intrinsic rhythm is detectable, temporary pacing withdrawal during CMR acquisition might be considered to assess myocardial inflammation.

Permanent pacemaker implantation requires comprehensive evaluation of these multifaceted factors. Following previous reports, we avoided implantation within 1 week of onset. We then waited for normalization of cardiac enzymes and improvement of cardiac function and assessed that both had largely normalized by day 21. To confirm that inflammation had subsided and to identify any unrecognized inflammation or damage, we considered additional imaging evaluation. Because CMR was not feasible, we confirmed inflammatory resolution through repeated endomyocardial biopsy. Based on multifaceted assessment using these combined modalities for irreversibility, we proceeded to permanent pacemaker implantation after comprehensive multidisciplinary evaluation.

## Conclusions

This case demonstrated a challenging clinical situation of lymphocytic myocarditis complicated by CAVB. Cardiac function and inflammatory findings completely normalized, and chronic-phase CMR revealed no LGE; however, conduction abnormalities alone persisted. Considering these findings comprehensively, determining the timing for permanent pacemaker implantation based on a single indicator is difficult. Beyond continuously monitorable parameters such as hemodynamics, cardiac enzymes, and wall motion, comprehensive evaluation of fluorodeoxyglucose positron emission tomography, CMR, and endomyocardial biopsy over time is essential for complex decision-making. This case emphasizes the importance of comprehensive evaluation when determining permanent pacemaker implantation timing in atrioventricular block complicating myocarditis.

## Funding Support and Author Disclosures

The authors have reported that they have no relationships relevant to the contents of this paper to disclose.

## Appendix

Video 1 Echocardiography on admission It revealed severe diffuse hypokinesis with an ejection fraction of 30%. The left ventricular wall was mildly thickened with no significant valvular abnormalities.

Video 2 Echocardiography on day 6 It revealed incomplete improvement of left ventricular function, but demonstrated improvement compared to admission.

Video 3 Echocardiography on day 21 It revealed nearly normalized left ventricular function, and wall thickness had normalized.

Video 4 Echocardiography in the chronic phase Cardiac function remained normal.
